# The Price of Explainability in Machine Learning Models for 100-Day Readmission Prediction in Heart Failure: Retrospective, Comparative, Machine Learning Study

**DOI:** 10.2196/46934

**Published:** 2023-10-27

**Authors:** Amira Soliman, Björn Agvall, Kobra Etminani, Omar Hamed, Markus Lingman

**Affiliations:** 1 Center for Applied Intelligent Systems Research School of Information Technology Halmstad University Halmstad Sweden; 2 Department of Research and Development Region Halland Halmstad Sweden; 3 Center for Primary Health Care Research Department of Clinical Sciences Malmö, Lund University Malmö Sweden; 4 Department of Molecular and Clinical Medicine Institute of Medicine Sahlgrenska Academy, University of Gothenburg Göteborg Sweden; 5 Department of Cardiology Institute of Medicine Sahlgrenska Academy, University of Gothenburg Göteborg Sweden

**Keywords:** readmission prediction, heart failure, machine learning, explainable artificial intelligence, deep learning, shallow learning

## Abstract

**Background:**

Sensitive and interpretable machine learning (ML) models can provide valuable assistance to clinicians in managing patients with heart failure (HF) at discharge by identifying individual factors associated with a high risk of readmission. In this cohort study, we delve into the factors driving the potential utility of classification models as decision support tools for predicting readmissions in patients with HF.

**Objective:**

The primary objective of this study is to assess the trade-off between using deep learning (DL) and traditional ML models to identify the risk of 100-day readmissions in patients with HF. Additionally, the study aims to provide explanations for the model predictions by highlighting important features both on a global scale across the patient cohort and on a local level for individual patients.

**Methods:**

The retrospective data for this study were obtained from the Regional Health Care Information Platform in Region Halland, Sweden. The study cohort consisted of patients diagnosed with HF who were over 40 years old and had been hospitalized at least once between 2017 and 2019. Data analysis encompassed the period from January 1, 2017, to December 31, 2019. Two ML models were developed and validated to predict 100-day readmissions, with a focus on the explainability of the model’s decisions. These models were built based on decision trees and recurrent neural architecture. Model explainability was obtained using an ML explainer. The predictive performance of these models was compared against 2 risk assessment tools using multiple performance metrics.

**Results:**

The retrospective data set included a total of 15,612 admissions, and within these admissions, readmission occurred in 5597 cases, representing a readmission rate of 35.85%. It is noteworthy that a traditional and explainable model, informed by clinical knowledge, exhibited performance comparable to the DL model and surpassed conventional scoring methods in predicting readmission among patients with HF. The evaluation of predictive model performance was based on commonly used metrics, with an area under the precision-recall curve of 66% for the deep model and 68% for the traditional model on the holdout data set. Importantly, the explanations provided by the traditional model offer actionable insights that have the potential to enhance care planning.

**Conclusions:**

This study found that a widely used deep prediction model did not outperform an explainable ML model when predicting readmissions among patients with HF. The results suggest that model transparency does not necessarily compromise performance, which could facilitate the clinical adoption of such models.

## Introduction

Unscheduled rehospitalization has garnered significant research attention due to its high cost and its association with unfavorable patient outcomes [1,2]. In particular, the rehospitalization of patients with heart failure (HF) has been a focal point of the investigation, given the alarmingly high unscheduled readmission rates, which recent studies have reported to be around 30% within 3 months of hospital discharge [3-6]. In the context of value-based care, health care providers are actively working to minimize readmission rates and enhance patient care outcomes. Reducing readmissions involves implementing measures both during a patient’s hospital stay and in the postdischarge phase to ensure adherence to care plans and optimize treatment [3].

Numerous risk factors have been identified as being related to high-risk readmissions in patients with HF. Specifically, there are correlations with serum potassium levels, N-terminal prohormone of brain natriuretic peptide (NT-proBNP), and suboptimal medication adherence [2,3,7]. Moreover, HF is accompanied by several significant comorbidities, including hypertension, diabetes, chronic kidney disease, and atrial fibrillation, all of which impact both the management and prognosis of the condition [2,3].

Machine learning (ML) techniques, especially deep learning (DL), have proven to be highly effective for readmission prediction tasks. This is because the data inputs involved are often intricate and may not be readily discernible by physicians [8-11]. DL methods, in particular, require substantial amounts of data and computational power to uncover latent patterns, eliminating the necessity for domain-specific knowledge. Nevertheless, DL models are often associated with limited explainability owing to their sheer complexity [12]. Conversely, the abundance of variables within electronic health care records (EHRs) poses a challenge for traditional ML methods, often referred to as shallow ML techniques. Consequently, the feature engineering step becomes essential for extracting meaningful features from raw data, often with the assistance of domain-specific knowledge.

Recently, shallow ML has exhibited the potential to surpass state-of-the-art DL techniques when accompanied by effective feature engineering [7-10]. Consequently, the shallow ML approach not only requires less training time but also yields discernible features. Model features would be more straightforward to grasp, offering greater opportunities to interpret the outcomes. Nevertheless, it is crucial that the feature engineering step is performed in collaboration with continuous feedback from clinical experts, ensuring the selection of pertinent variables for modeling the prediction task. Clinicians provide essential guidelines on which data hold clinical significance and how to classify those data. This clinical guidance is crucial for making decisions, such as determining which laboratory values to prioritize and whether transforming continuous values into categories, such as high, normal, or low, can enhance the performance of the ML model.

ML has the potential to aid clinicians in the management of patients with HF at the point of discharge by predicting those at an elevated risk of readmission [13]. However, a lingering question is how to quantify the practical utility of these models in actual clinical practice. Previous studies have demonstrated the potential applicability of their developed models in achieving cost savings [14]. Nonetheless, when it comes to integrating a model into the clinical workflow, it is not just the model’s discrimination ability that holds significance, but also its applicability in real clinical scenarios. To instill confidence in clinicians, it is essential to provide interpretability of the model results, elucidating the individual variables that influence the model’s decisions. This enables clinicians to verify the clinical implications of patient conditions [15].

This study aims to explore the potential of using ML to forecast the risk of readmission due to HF deterioration within 100 days after discharge. This investigation involves a comparison between shallow and deep models applied to real-world health care data. Furthermore, our objective is to enhance the interpretability of these models to ensure they are clinically reliable and actionable.

## Methods

### Data Source

This study is based on retrospective data from the Regional Health Care Information Platform in Region Halland, an integrated care system located in southwestern Sweden [16]. The system includes data from both the primary and secondary health care levels, including all prescribed medications, clinical examination results (eg, laboratory assessments and radiological examinations), and care delivery resources. Diagnoses (including procedures) and medications were presented according to standard schemas: International Classification of Diseases, revision 10, Sweden (ICD-10-SE) and Anatomical Therapeutic Chemical (ATC) classification system codes, respectively.

### Ethics Considerations

The study was approved by the Swedish Ethical Review Board, Stockholm Department 2 Medicine (registration number 2020-00455). Informed consent was waived because this was a retrospective study that the Swedish Ethical Review Board approved. All the methods in this study were performed in accordance with relevant guidelines and regulations.

### Study Population

The cohort for this study comprised individuals who had been diagnosed with HF based on ICD-10-SE codes (I11.0, I42, I43, and I50; see Table S4 in Multimedia Appendix 1), were residents receiving care within the Region Halland, and met specific inclusion criteria (ie, aged ≥40 years and had experienced at least one hospital admission after their HF diagnosis between January 1, 2017, and December 31, 2019). We considered all-cause hospitalization. In our study, for each admission within the cohort, we took into account all of the patient’s prior admissions within a 5-year period preceding the current admission (referred to as the lookback period). It is important to note that these previous admissions were not treated as events within our study; instead, they were exclusively used as historical data.

We excluded the following from our analysis: hospitalizations that occurred before the patient’s initial HF diagnosis, hospitalizations of patients younger than 40 years of age at the index admission, hospitalizations in which patients passed away before discharge, and hospitalizations in which patients passed away within 100 days following discharge. Additionally, hospitalizations with a length of stay exceeding 31 days were excluded, as they exhibited a higher likelihood of being influenced by multiple coexisting diseases, as well as factors such as frailty and other medical conditions. The study cohort consisted of 6040 patients, with 2728 (45.02%) being women. These patients collectively contributed to a total of 20,598 hospital admissions. However, after implementing our exclusion criteria, as outlined in Figure S1 in Multimedia Appendix 1, we considered 15,612 (75.79%) of these admissions for our analysis.

### Patient Variables and Data Definitions

We collected variables from the Regional Health Care Information Platform that can have a significant influence on readmission prediction as shown in Tables 1-4. The variables considered were patient demographics, comorbidities, medications, and laboratory results. The choice of variables was made through a collaborative process involving both clinical expertise and data science knowledge. For example, we selected comorbidities including hypertension, chronic kidney disease, and atrial fibrillation. We also applied the Charlson Comorbidity Index (CCI) to compute a score associated with different medical comorbid conditions, such as liver disease, diabetes, and obstructive pulmonary disease [17,18]. These comorbidity variables consider the complete patient history, including all visits documented in the EHR system, encompassing specialist and primary care visits. We used ICD-10-SE codes to calculate these comorbidity features, and additional information can be found in Table S2 in Multimedia Appendix 1.

**Table 1:** Baseline demographics and comorbidities.

Variables			Total (N=15,612)		Not readmitted (n=10,015)		Readmitted (n=5597)		*P* value
**Demographics at index**									
	Sex: Female, n (%)	6905 (44.23)		4472 (44.65)		2433 (43.47)		.34	
	Age (years), mean (SD)	79.1 (10.4)		79 (10.5)		79.3 (10.3)		.13	
	Duration of heart failure when admitted (days), mean (SD)	1149.9 (1036.6)		1133.2 (1032.3)		1179.9 (1043.5)		<.001	
**Comorbidities, n (%)**									
	Hypertension	12,948 (82.94)		8219 (82.07)		4729 (84.49)		.11	
	Ischemic heart disease	8275 (53.00)		5132 (51.24)		3143 (56.16)		<.001	
	Cerebrovascular insult	3878 (24.84)		2397 (23.93)		1481 (26.46)		.002	
	Valvular heart disease	4303 (27.56)		2563 (25.59)		1740 (31.09)		<.001	
	Peripheral artery disease	1470 (9.42)		863 (8.62)		607 (10.85)		<.001	
	Atrial fibrillation	10,001 (64.06)		6047 (60.38)		3954 (70.64)		<.001	
	Diabetes mellitus	4828 (30.92)		2944 (29.40)		1884 (33.66)		<.001	
	Chronic obstructive pulmonary disease	3727 (23.87)		2088 (20.85)		1639 (29.28)		<.001	
	Chronic kidney disease	6007 (38.48)		3429 (34.24)		2578 (46.06)		<.001	
Charlson Comorbidity Index, mean (SD)			3.0 (1.9)		2.7 (1.9)		3.5 (1.8)		<.001

**Table 2:** Clinical characteristics^a^.

Variables			Total (N=15,612)		Not readmitted (n=10,015)		Readmitted (n=5597)		*P* value
**N-terminal prohormone of brain natriuretic peptide, n (%)**									
	HF^b^ likely	4439 (28.43)		2494 (24.90)		1945 (34.75)		<.001	
	Gray zone for HF	1261 (8.08)		839 (8.38)		422 (7.54)		.08	
	HF unlikely	222 (1.42)		169 (1.69)		53 (0.95)		<.001	
**Sodium, n (%)**									
	High (value>145)	359 (2.30)		190 (1.90)		169 (3.02)		<.001	
	Normal (137≤value≤145)	8730 (55.92)		5539 (55.31)		3191 (57.01)		.17	
	Low (<137)	2171 (13.91)		1297 (12.95)		874 (15.62)		<.001	
**Potassium^c^ (mmol/L), n (%)**									
	High (value>4.7)	940 (6.02)		549 (5.48)		391 (6.99)		<.001	
	Normal (3.5≤value≤4.7)	11,796 (75.56)		7497 (74.86)		4299 (76.81)		.18	
	Low (value<3.5)	1236 (7.92)		773 (7.72)		463 (8.27)		.24	
**Ferritin <100 ng/mL, n (%)**									
	Abnormal	606 (3.88)		349 (3.48)		257 (4.59)		<.001	
	Normal	1109 (7.10)		668 (6.67)		441 (7.88)		.007	
**Estimated glomerular filtration rate, n (%)**									
	Extreme (value<30)	2437 (15.61)		1305 (13.03)		1132 (20.23)		<.001	
	Abnormal (30≤value<60)	6946 (44.49)		4444 (44.37)		2502 (44.70)		.77	
	Normal (60≤value)	4358 (27.91)		2937 (29.33)		1421 (25.39)		<.001	

^a^Unreported laboratory values were not included in the statistics presented.

^b^HF: heart failure.

^c^Some cases during 2017 were evaluated with 3.2 as the starting range to have a normal value. This applies to the low range as well.

**Table 3:** Heart failure treatment.^a^

Variables	Total (N=15,612)		Not readmitted (n=10,015)		Readmitted (n=5597)		*P* value	
	Discharge	Medication history within 1 year	Discharge	Medication history within 1 year	Discharge	Medication history within 1 year	Discharge	Medication history within 1 year
β-blockers, n (%)	7915 (50.70)	9063 (58.05)	5228 (52.20)	5755 (57.46)	2687 (48.01)	3308 (59.10)	<.001	.20
Angiotensin-converting enzyme inhibitors, n (%)	3270 (20.95)	4291 (27.49)	2297 (22.94)	2784 (27.80)	973 (17.38)	1507 (26.93)	<.001	.32
Angiotensin receptor blockers, n (%)	1979 (12.68)	2857 (18.30)	1392 (13.90)	1877 (18.74)	587 (10.49)	980 (17.51)	<.001	.08
Angiotensin receptor neprilysin inhibitors, n (%)	302 (1.93)	390 (2.50)	204 (2.04)	241 (2.41)	98 (1.75)	149 (2.66)	.22	.33
Mineralocorticoid receptor antagonists, n (%)	3764 (24.11)	4408 (28.23)	2401 (23.97)	2544 (25.40)	1363 (24.35)	1864 (33.30)	.64	<.001
Loop diuretics, n (%)	7264 (46.53)	8411 (53.88)	4588 (45.81)	5080 (50.72)	2676 (47.81)	3331 (59.51)	.08	<.001
Digoxin, n (%)	1110 (7.11)	1491 (9.55)	733 (7.32)	901 (9.00)	377 (6.74)	590 (10.54)	.19	.003
SGLT-2^b^, n (%)	127 (0.81)	182 (1.17)	102 (1.02)	134 (1.34)	25 (0.45)	48 (0.86)	<.001	.008

^a^The statistics are based on prescriptions documented in the system. There is a possibility that patients already have the medication and have no need to get new prescriptions at discharge (ie, medication history within 1 year>discharge).

^b^SGLT-2: sodium-glucose cotransporter-2.

**Table 4:** Administration features and hospitalization type.

Variables		Total (N=15,612)	Not readmitted (n=10,015)	Readmitted (n=5597)	*P* value
**Administration features (lookback period=5 years)**					
	Number of all-cause hospitalizations, mean (SD)	5.7 (7.8)	4.4 (5.5)	8.1 (10.3)	<.001
	Number of emergency visits (in the last 6 months), mean (SD)	2.3 (2.3)	1.9 (1.8)	3.1 (3.0)	<.001
	Number of procedures per admission, mean (SD)	2.3 (2.7)	2.4 (2.7)	2.3 (2.6)	.02
	Total length of stay during the lookback period per patient (days), mean (SD)	26.0 (32.7)	20.0 (24.5)	36.8 (41.6)	<.001
**Hospitalization type (index admission)**					
	Length of stay for index admission, mean (SD)	5.2 (5.2)	5.0 (5.1)	5.5 (5.2)	<.001

We collected the most recent results of specific laboratory tests conducted before discharge, including NT-proBNP [19], sodium, potassium, ferritin, and estimated glomerular filtration rate. Notably, NT-proBN*P* values were categorized into normal, gray zone, and elevated ranges [17]. The rest of the laboratory values were categorized into normal and abnormal ranges according to international clinical recommendations (eg, extreme, low, and high), focusing on extracting the correlations between abnormal laboratory values and readmission risk. For the missing laboratory values, we adopted the assumption that they were within the normal range, a methodology consistent with previous studies [20,21]. Furthermore, we gathered information about medications prescribed at the time of discharge, including β-blockers, renin-angiotensin-aldosterone system inhibitors, mineralocorticoid receptor antagonists, loop diuretics, and sodium-glucose cotransporter-2 (SGLT-2) inhibitors. Detailed specifications and ATC codes can be found in Table S3 in Multimedia Appendix 1. In addition, we considered the patient’s medication history by introducing variables to indicate whether each medication had been prescribed at discharge within the previous year for each admission.

We also incorporated a few administrative features related to hospital utilization during the lookback period. These included the total length of stay for inpatient care, the count of outpatient visits (including hospital-associated and primary health care visits), and the number of all-cause hospitalizations. Tables 1-4 display the baseline characteristics of the HF cohort and the engineered features used for training the ML models. It is worth noting that the same patient cohort has been previously investigated, and the clinical variables used in this study have been identified as being linked to the estimation of 100-day readmission risk [22].

The target label, termed “readmission risk,” is defined as a binary variable. A default value of 0 signifies low risk. However, this label is set to 1 if a patient experiences an unplanned admission within 100 days following their discharge. It is important to note that cases in which patients were discharged to another health care facility or in-hospital transfers were not regarded as unplanned readmissions.

### Modeling Strategies

We conducted a comparison between the 2 modeling approaches. Our initial model was constructed using CatBoost (Yandex), representing a shallow model developed through traditional ML techniques. CatBoost uses gradient boosting decision trees, renowned for their capacity to handle both categorical and continuous features [23]. The CatBoost model generates predictions by using a series of decision trees, rendering it a more explainable model. By contrast, deep models, specifically recurrent neural networks such as long short-term memory (LSTM) networks [24], have garnered significant attention due to their ability to model the sequential nature inherent in EHRs [7,10,21]. Hence, our second model is constructed as an LSTM network, as depicted in Figure S2 in Multimedia Appendix 1. The LSTM model is designed to learn intricate nonlinear associations between the model variables and outcomes, necessitating the establishment of a surrogate model for providing explanations. In this research, we opted for the LSTM network architecture for DL. Nonetheless, it is worth noting that sequential information regarding patient visits can also be effectively modeled using other recurrent neural network architectures [7].

**Figure 1:**
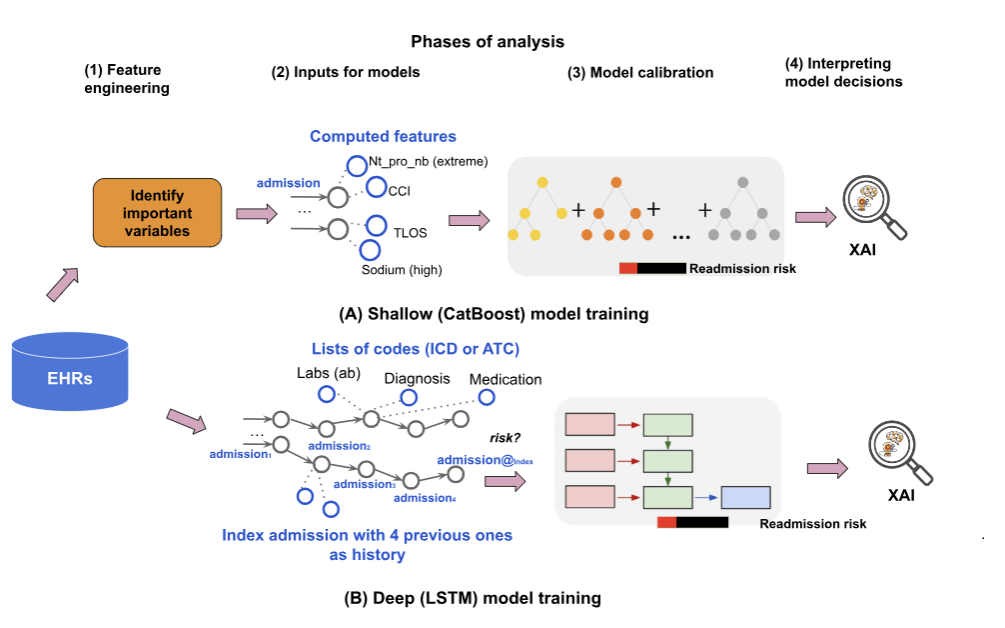
Data preparation and modeling strategies. The 2 modeling pipelines adopted for (A) the shallow model and (B) the deep learning model. We have 4 phases of analysis: (1) features engineered to prepare values of important variables to be used for the shallow model; (2) preparing input for different models, a set of numeric values named engineered features for the shallow model, or a sequence of 5 visits as used for the deep model represented as a mix of codes (ICD or ATC) and numeric values; (3) model calibration for training the different models; and (4) providing explanations for model decisions. ATC: Anatomical Therapeutic Chemical; CCI: Charlson Comorbidity Index; EHR: electronic health record; ICD: International Classification of Diseases; LSTM: long short-term memory; TLOS: total length of stay; NT-proBNP: N-terminal prohormone of brain natriuretic peptide; XAI: Explainable Artificial Intelligence.

Figure 1 illustrates the various levels of analysis conducted in this study. As we used 2 distinct data modeling strategies, we organized the data into 2 different formats. For the shallow model, the input data were structured as a single record containing a list of engineered features, computed based on predefined variables outlined in Tables 1-4. Conversely, the input for the DL model comprised raw structured EHR-based data for a sequence of 5 admissions, consisting of the index admission and the 4 prior ones. For each of these admissions, we treated the associated diagnoses as a list of ICD-10-SE codes. Similarly, we regarded procedures, medications, and abnormal laboratory test results as lists in the DL model. However, the shallow model involved an additional preliminary step, the feature engineering phase, in which we computed the values of engineered features. The primary objective of this phase is to convert raw data into a collection of computed features, which are utilized as input for the CatBoost model. Subsequently, we progress through the next 3 phases for both models: input provision, model calibration, and the extraction of explanations for model decisions.

Apart from the models we developed, we also assessed the LACE (Length of stay, Acuity of index admission, CCI, and number of emergency department [ED] visits in the last 6 months) index as an evaluation tool. The LACE index is a tool used for predicting unscheduled readmission, utilizing readily available clinical predictors that can be calculated before a patient’s discharge [25]. The LACE index is formulated using 4 variables to estimate the risk of either death or non-elective readmission following a patient’s discharge from the hospital. These variables are the length of stay, acuity of the index admission, the CCI, and the count of ED visits within the last 6 months [26].

### Model Explainability

We used the Shapley Additive Explanations (SHAP) technique to offer insights into model decisions by revealing the most important features for readmission prediction. The SHAP functions as a model-agnostic explanation tool, providing both local and global explanations. The SHAP uses coalitional game theory and offers a model-agnostic approach for computing both the overall behavior of the model and local explanations for specific readmissions [27]. The global explanations furnish a roster of critical features that either contribute to or detract from the risk of readmission. The technique also assigns a score to each prediction through the calculation of additive feature importance. This score can highlight the risk factors that are either positively or negatively influencing the readmission risk for a specific admission. Additionally, clinicians evaluated the explanations offered by each model from a clinical perspective. In particular, clinicians provided feedback on the most critical features identified by SHAP in the context of their clinical assessment of patient conditions. They assessed whether these features could serve as indicators or warnings of undesirable outcomes.

### Statistical Analysis

We divided the data into 2 segments. The first subset, spanning from January 1, 2017, to August 31, 2019, consisting of 13,800/15,612 admissions (88.39%), was used for model training. The second, more recent subset, spanning from September 1, 2019, to December 31, 2019, containing 1812/15,612 admissions (11.61%), was reserved for holdout testing. We fine-tuned the hyperparameters of each model using the grid search method, which conducts a thorough exploration of a predefined set of hyperparameter combinations. To train the models, we implemented a stratified 10-fold cross-validation approach, where the training data were subdivided into 10 segments. In each iteration, a fold consisting of randomly chosen 90% (12,420/13,800) admissions of the training data was used for model training, while the remaining admissions (1380/13,800, 10%) were used for validation. This process was repeated 10 times, with a different 10% of the data reserved for validation in each iteration. We used a sensitivity-based training strategy to optimize the sensitivity of the trained models, making them particularly relevant in clinical operations. Of the 10-fold cross-validation training process, we selected the model that achieved the highest sensitivity while maintaining a minimum specificity of 50%.

The assessment of predictive model performance is founded on widely used performance metrics, including sensitivity, specificity, *F*_1_-score, receiver operating characteristic curves, the area under the receiver operating characteristic curve (AUC), and the area under the precision-recall curve, all of which are reported with 95% CIs. Additionally, we calculated the number needed to screen, the number needed to diagnose, and the number needed to predict [28].

## Results

### Patient Variables and Data Definitions

The features of admissions, encompassing both unscheduled readmissions and planned admissions within the following 100 days after discharge, are detailed in Tables 1-4. As indicated, the instances of readmitted patients exhibited a notably higher prevalence of comorbidities in comparison to those cases that did not result in readmission.

### Model Evaluation

Table 5 presents the performance achieved by the models, with the characteristics of the training and holdout test cohorts detailed in Table S1 in Multimedia Appendix 1. It is worth noting that CatBoost outperformed LSTM in both the validation and holdout test sets, achieving superior sensitivity and specificity. The sensitivity-based training approach led to an increased number of false positives, which are misclassified admissions labeled as unplanned readmissions, in both models, consequently resulting in lower specificity. Concerning the other metrics, the performance of both models is nearly identical. In both cases, the numbers needed to screen/diagnose/predict were approximately 4-5. Notably, the models we developed exhibited superior performance when compared with the LACE index. We used a threshold of 10 to identify high-risk readmissions based on the LACE scores index [26].

**Table 5:** Validation and testing accuracy reported by different models.^a^

Metric		Deep (LSTM^b^) model	Shallow (CatBoost) model	LACE^c^ score
**Validation^d^**				
	Sensitivity, mean (SD)	0.83 (0.019)	*0.84* (0.016)	0.38 (0.026)
	Specificity^e^	0.51 (0.022)	0.52 (0.025)	*0.77* (0.017)
	AUC^f^	0.67 (0.008)	*0.68* (0.014)	0.57 (0.015)
	*F*_1_-score	0.61 (0.017)	*0.62* (0.021)	0.42 (0.026)
	AUPRC^g^	0.69 (0.014)	*0.70* (0.016)	0.54 (0.026)
**Holdout^h^**				
	Sensitivity (95% CI)	0.78 (0.72-0.78)	*0.83* (0.79-0.86)	0.35 (0.31-0.38)
	Specificity^e^ (95% CI)	0.53 (0.53-0.58)	0.50 (0.47-0.52)	*0.78* (0.76-0.80)
	AUC (95% CI)	0.65 (0.63-0.67)	*0.66* (0.64-0.68)	0.56 (0.54-0.58)
	*F*_1_-score (95% CI)	0.58 (0.55-0.61)	*0.60* (0.57-0.63)	0.39 (0.36-0.42)
	AUPRC (95% CI)	0.66 (0.63-0.68)	*0.68* (0.66-0.70)	0.51 (0.48-0.54)
	Number needed to screen (95% CI)	4.33 (3.43-4.95)	4.49 (3.81-5.68)	3.84 (3.27-4.59)
	Number needed to diagnose (95% CI)	3.24 (2.81-3.77)	3.06 (2.77-3.54)	7.90 (5.90-12.14)
	Number needed to predict (95% CI)	3.50 (3.04-4.15)	3.20 (2.89-3.62)	6.79 (5.08-10.65)

^a^Italicized values represent the best model over that metric.

^b^LSTM: long short-term memory.

^c^LACE: Length of stay, Acuity of index admission, CCI (Charlson Comorbidity Index), and number of ED (emergency department) visits in the last 6 months.

^d^The mean and SD of different metrics obtained during 10-fold cross-validation training are presented.

^e^The reason behind lower specificity ratios for both LSTM and CatBoost models is due to sensitivity-based training; hence a lower specificity (minimum of 0.50) is accepted to reach higher sensitivity.

^f^AUC: area under the receiver operating characteristic curve.

^g^AUPRC: area under the precision-recall curve.

^h^The performance on the holdout test cohort for the model that achieved the highest sensitivity with a minimum specificity of 50% after 10-fold cross-validation training is reported.

As aforementioned, we opted for sensitivity over specificity to be relevant in clinical operations, thus, our models generate more false positives than the LACE index. The LACE index is not trainable compared with the proposed CatBoost and LSTM models, yet it is commonly used to estimate readmission risks in general. However, a study on patients with HF showed that high LACE scores were associated with higher rates of ED revisits after hospital discharge but they had less accuracy in predicting readmissions [25].

The lower specificity ratios observed for both LSTM and CatBoost models are attributed to the sensitivity-based training approach we used. This approach prioritizes achieving a higher sensitivity, and as a result, accepting a lower specificity (with a minimum of 0.50) is part of the strategy to achieve this goal.

### Model Explainability

**Figure 2:**
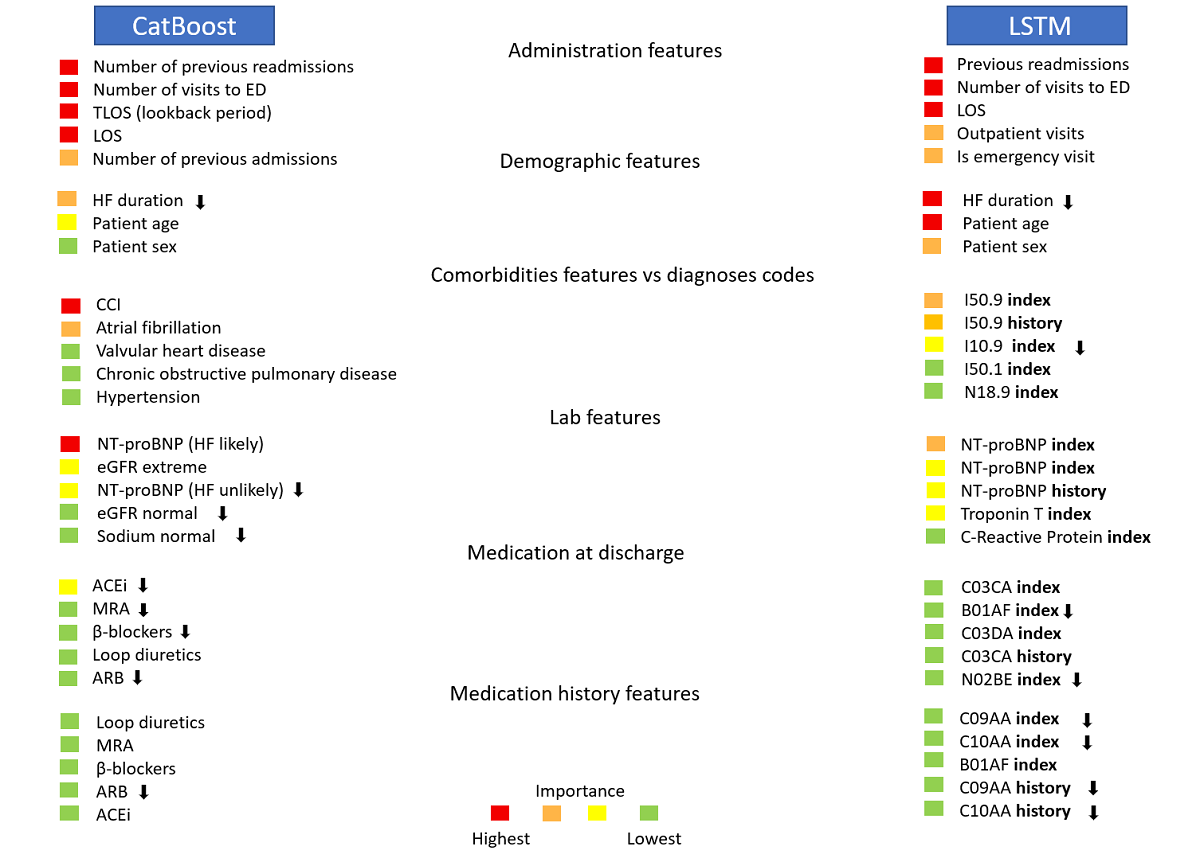
Global explanations: list of important variables for the shallow (CatBoost) and deep (LSTM) models. The color reflects the level of importance of each variable out of 4 levels computed from the SHA*P* values assigned to variables. The arrows indicate those variables that are pushing against readmission risk (negatively correlated). ACEi: angiotensin-converting enzyme inhibitor; ARB: angiotensin receptor blocker; CCI: Charlson Comorbidity Index; ED: emergency department; eGFR: estimated glomerular filtration rate; HF: heart failure; ICD-10-SE: International Classification of Diseases, revision 10, Sweden; LOS: length of stay in this admission; LSTM: long short-term memory; MRA: mineralocorticoid receptor antagonist; NT-proBNP: N-terminal prohormone of brain natriuretic peptide; SHAP: Shapley Additive Explanations; TLOS: total length of stay at hospital during the lookback period. For medication codes and diagnostic ICD-10-SE, full names are available at Tables S3 and S4 in Multimedia Appendix 1, respectively.

Figure 2 provides an overview of the variables of importance identified by the CatBoost and LSTM models, categorized into several main groups. Given that we used 2 distinct input formats for these models, our interest lies in understanding the commonalities and disparities in what they have identified. Both models concurred in identifying the most critical variables for predicting readmission. These variables included a higher number of previous readmissions, emergency admissions, longer lengths of stay, and age. By contrast, the history of medications was assigned a lower level of importance by both models. Furthermore, both models highlighted extreme values of NT-proBNP as an influential variable pushing toward readmission. Additionally, both models indicated that patients prescribed loop diuretics at discharge were at a higher risk of readmission. However, CatBoost identified angiotensin-converting enzyme inhibitors, mineralocorticoid receptor antagonists, β-blockers, and angiotensin receptor neprilysin inhibitor treatments at discharge as factors associated with a lower risk of readmission.

For the CatBoost model, the CCI was identified as the most critical feature. This feature, however, was not available for the LSTM model. Conversely, the LSTM model placed greater importance on diagnosis codes compared with other clinical information such as abnormal laboratory test results and medication codes. Interestingly, in both models, patient age was associated with an increased risk of readmission, while the duration of HF appeared to reduce this risk. You can find the top 28 features highlighted by both models in Figures S3 and S4 in Multimedia Appendix 1.

**Figure 3:**
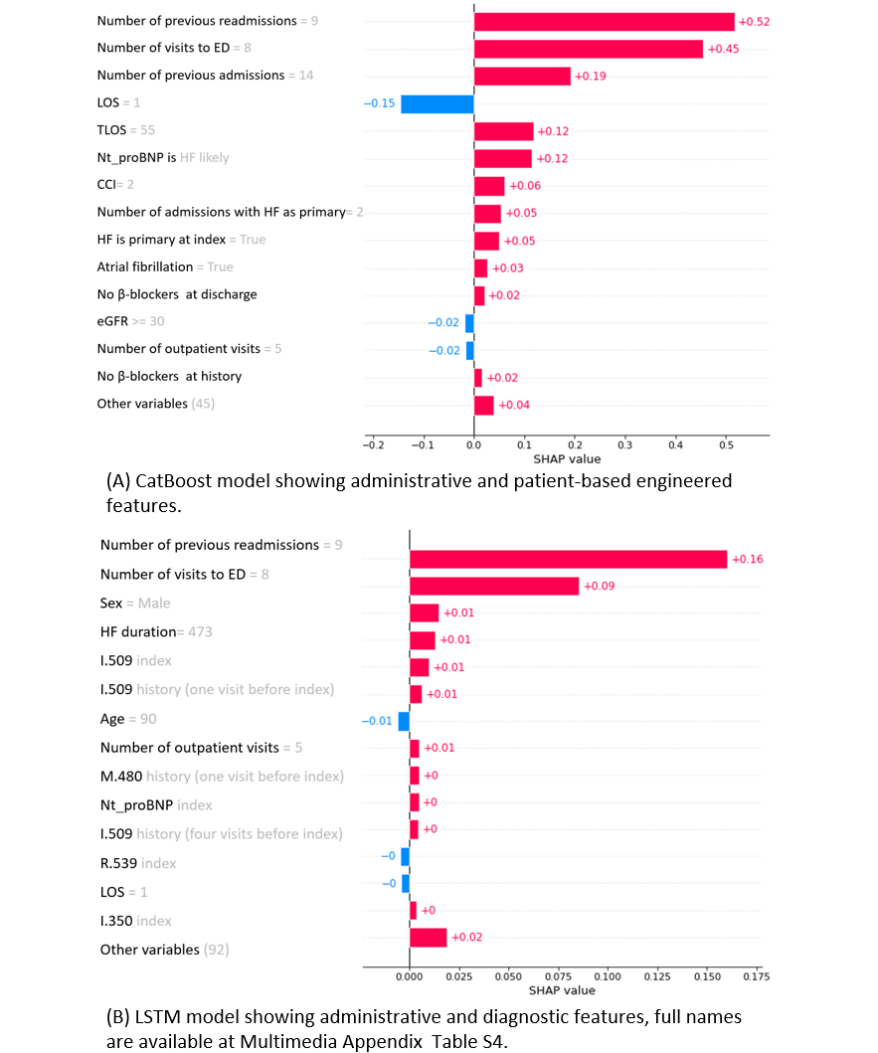
Local explanations for a correctly classified “Readmitted” case. (A) CatBoost (shallow model) and (b) LSTM (deep model). The red values indicate the importance of that feature toward readmission risk and the blue ones against it. CCI: Charlson Comorbidity Index; eGFR: estimated glomerular filtration rate; HF: heart failure; ICD-10-SE: International Classification of Diseases, revision 10, Sweden; LOS: length of stay; LSTM: long short-term memory; NT-proBNP: N-terminal prohormone of brain natriuretic peptide; TLOS: total length of stay.

Local explanations are valuable for enabling health care professionals to comprehend the rationale behind model predictions and make informed decisions. In Figure 3, local explanations are provided for a particular admission where both models correctly classified it as a high-risk readmission. In the case presented, the 2 most important features were identical in both models: the number of previous unscheduled readmissions and the number of ED visits. Additionally, both models found that the presence of chronic kidney disease and abnormal laboratory test values of NT-proBNP (indicative of HF) contributed to an increased risk of readmission. Conversely, a short length of stay, specifically 1 day in this instance, exerted a negative impact on the risk of readmission. Additional examples, where the models disagree on the target label, can be found in Figures S5 and S6 in Multimedia Appendix 1.

## Discussion

### Principal Findings

Our primary motivation for this study is to develop models that can effectively adapt to patterns within patients with HF data. Given the absence of prior research demonstrating that ML models can substantially reduce readmission, our study focuses on the comparison of shallow and DL models in the context of patients with HF data. This study encompassed a large HF cohort, with a readmission rate of 5597/15,612 (35.85%) per year, which aligns with findings from comparative studies [3,6,22]. Notably, the more interpretable shallow model exhibited superior performance compared with the deep model, and both models significantly outperformed conventional scores in terms of the numbers needed to screen for identifying readmission within 100 days. Consequently, this study does not suggest that achieving enhanced prediction performance necessitates sacrificing model transparency.

The results indicate that the shallow model, while more interpretable, performs as effectively as the deep model. However, it is important to note that the shallow model necessitates a feature engineering process to derive useful features from raw EHR data. By contrast, the input to the deep model closely resembles the original EHR data, enabling a comparison of the patterns learned from raw data against those generated by engineered features calculated with the guidance of expert knowledge. Interestingly, the explainability results reveal that there is an overlap in the most critical features identified by both models.

Good explanations can serve as an advantage in building trust among clinicians and guiding interventions. Understanding the features that influenced the prediction is crucial, as previous reports have emphasized the importance of comprehending the models to establish trust in them [14,15]. The selection of variables used for model training was made to support generalizability and the potential transfer of the model to new settings. ICD-10-SE codes, ATC codes, and standard laboratory values are expected to be available in most acute care venues, enhancing the applicability of the model.

### Model Explainability and Clinical Insights

In addition to clinically informative variables, administrative features exhibit significant importance in both models, consistent with findings from prior studies [8,11,29]. It appears that a history of previous hospital admissions, readmissions, and visits to the ED are associated with an elevated risk of readmission. Both models underscore the connection between patients with frequent inpatient episodes, often indicative of a high disease burden, and features that are not easily altered, such as age and comorbidity. Nevertheless, these features serve as crucial warning signals that should be considered when planning hospital discharge. As a result, the feature engineering process in this study was continually refined to encompass additional clinically relevant features. Given that previous readmissions and ED visits are robust indicators of potential new events, the factors contributing to past unplanned health care encounters could serve as key targets for action. Consequently, further investigation into these factors, with an emphasis on causal inference, is warranted to improve patient outcomes and reduce readmission rates.

Previous studies have demonstrated the positive impact of HF medication in preventing readmission [6]. In addition to medical treatment, factors such as continuity and availability of care are valuable tools for these patients. Insights revealing weak associations, such as the finding that guideline-compliant pharmaceutical treatment is only weakly linked to readmission risk, should emphasize the importance of focusing on nonmedical strategies to prevent readmissions alongside optimizing drug regimens, with an emphasis on drugs known to reduce readmission rates [30-32]. We should also not overlook previous findings that sodium and potassium levels, as well as NT-proBNP, are associated with the outcomes, as confirmed in this report. Moreover, the use of β-blockers and other first-line treatments can have an impact on patient outcomes. These factors should continue to be considered in patient care and intervention strategies [2,3,6,7].

Our focus on readmission serves a dual purpose: it acts as an indicator of health outcomes and, simultaneously, as an encounter that contributes to resource utilization, potentially conflicting with other health care priorities. The need to screen (and subsequently intervene with) approximately 4.5 patients to identify and target 1 potentially preventable readmission should be recognized as a compelling reason for further interventional studies, where the cost of the intervention is assessed. One potential intervention approach could involve care planning tailored to the identified drivers of readmission probability as determined in this study.

### Limitations

In this study, we did not encompass aspects of care beyond hospitals and primary health care. As readmissions were only weakly associated with pharmaceutical treatment and several other predictive factors were not easily amenable to intervention, strategies to reduce readmissions may need to concentrate on home care and enhancing patient comfort. However, it is important to note that these aspects were not within the scope of this study.

Moreover, the number of patients on SGLT-2 inhibitors in our study was too small to draw definitive conclusions about their impact on readmissions. However, other studies suggest that this medication warrants further investigation in real-world health care settings. It is important to emphasize that the effectiveness of treatment with drugs such as SGLT-2 inhibitors is not questioned according to guidelines and remains one of the major tools for improving patient outcomes.

### Comparison With Prior Work

A broad spectrum of predictive modeling methods, including statistical approaches and conventional ML and DL methods, have been used to tackle the readmission prediction problem [14]. There is a prevailing trend in the literature toward the utilization of traditional statistical and conventional ML models. These models are favored because they offer greater transparency, are easier to understand, and have been shown to outperform DL models in certain contexts [8,9,25]. Prior studies have often concentrated on comparing the predictive performance of different models, often neglecting to investigate their interpretability and practical usefulness in real clinical settings. However, with the rapid advancements in explainable artificial intelligence and the growing adoption of model-agnostic interpretation techniques, even complex DL models can offer both local and global explanations, as demonstrated in this study.

### Conclusions

In clinical prediction models, there can be a trade-off between precision and explainability. When models exhibit similar prediction performance, the less opaque or more interpretable model is often favored. In the presented case, the shallow ML model performs at a similar level to a deeper model and significantly outperforms traditional scoring methods. Achieving explainability requires the collaborative effort of clinicians through feature engineering. However, it is worth noting that many of the features driving the predictions in this study may not be easily actionable. Enhancing compliance with treatment guidelines can lead to improvements, but for more substantial impacts, exploring new drugs and interventions beyond the scope of this study should also be considered.

## Appendix

Multimedia Appendix 1 Data analysis and classification codes.

## Abbreviations

ATC: Anatomical Therapeutic Chemical
AUC: area under the receiver operating characteristic curve
AUPRC: area under the precision-recall curve
CCI: Charlson Comorbidity Index
DL: deep learning
ED: emergency department
EHR: electronic health record
HF: heart failure
ICD-10-SE: International Classification of Diseases, revision 10, Sweden
LACE: Length of stay, Acuity of index admission, CCI, and number of ED visits in the last 6 months
LSTM: long short-term memory
ML: machine learning
NT-proBNP: N-terminal prohormone of brain natriuretic peptide
SGLT-2: sodium-glucose cotransporter-2
SHAP: Shapley Additive Explanations

## Data Availability

The real-world data used in the analysis are protected to comply with Swedish integrity-protecting regulations, and thus, data cannot be accessible to the public due to the requirements of the Swedish Health and Medical Services Act.
