# Supplementary material for: The Price of Explainability in Machine Learning Models for 100-Day Readmission Prediction in Heart Failure: Retrospective, Comparative, Machine Learning Study
Source: J Med Internet Res. 2023 Oct 27;25:e46934. doi: 10.2196/46934 (PMC10638630; doi:10.2196/46934)
Supplement: Multimedia Appendix 1 [file jmir_v25i1e46934_app1.docx]

**Appendices**

**A Inclusion and Exclusion Criteria**

**Figure S1**. Cohort inclusion and exclusion criteria.

**B Model Development**

**Figure S2**. Architecture of deep model developed for readmission prediction.

**Table S1**. Baseline characteristics—training and holdout test cohorts.

**C Specification of Medical Codes**

**Table S2**. Specification of diseases used as comorbidity features according to ICD-codes.

**Table S3**. Specification of treatment according to ATC-codes.

**Table S4**. Specification ICD-10-SE codes reported by LSTM explanation model.

**D SHAP Explanations**

**Figure S3**. SHAP summary for Shallow (CatBoost) model showing top 30 features.

**Figure S4**. SHAP summary for Deep (LSTM) model showing top 30 features.

**Figure S5**. Local explanations for a readmission case correctly classified by Shallow (CatBoost) model.

**Figure S6**. Local explanations for a readmission case correctly classified by Deep (LSTM) model.

**A Inclusion and Exclusion Criteria**

The retrospective data were collected from the regional healthcare information platform (RHIP) in Region Halland, Sweden. Our inclusion criteria identify HF patients >40 years old who were hospitalized at least once between 2017 and 2019. Figure A.1 shows our exclusion criteria after collecting admissions satisfying inclusion conditions, we excluded all hospitalizations before the first HF diagnosis, hospitalizations for patients <40 years of age at index admission, hospitalizations where patients died before discharge, and hospitalizations where patients died within 100-days of discharge. Hospitalizations with a length of stay longer than 31-days were also excluded.

**
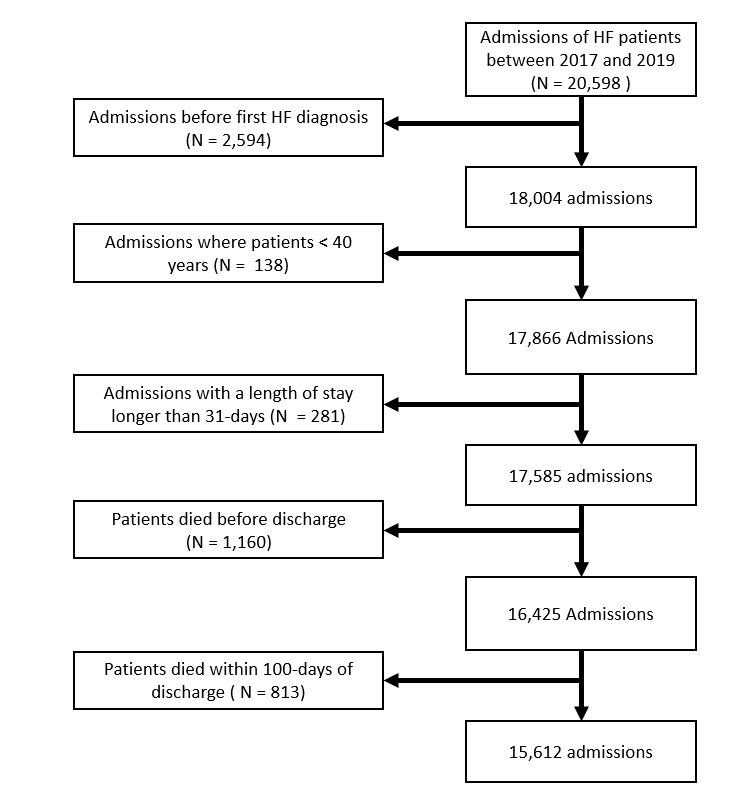
**

Figure S1: Cohort inclusion and exclusion criteria

**B Model Development**

**Shallow (CatBoost) Model**

CatBoost is a gradient boosting technique that uses ordered boosting and a novel categorical features processing algorithm to overcome the prediction shift issue that exists in other gradient boosting algorithms implementations. In this study we used the CatBoost (available via catboost.ai) implemented with python package V1.0.4.

**Deep (LSTM) Model**


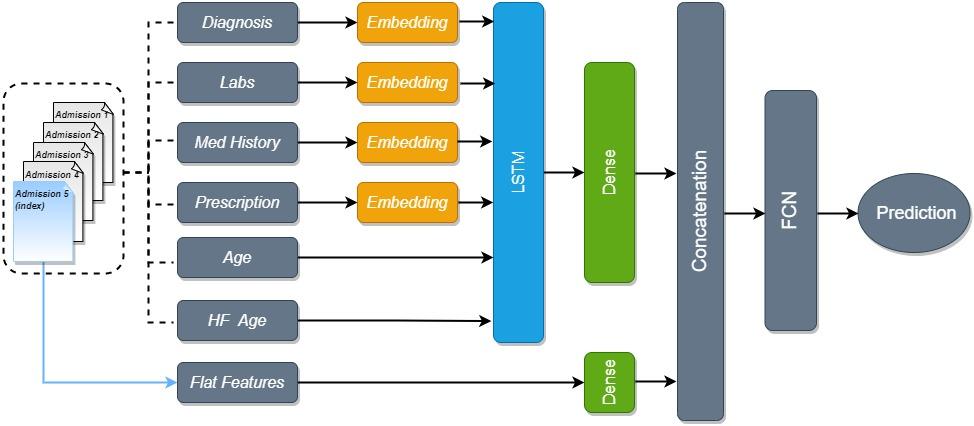


Figure S2: Architecture of developed deep model using LSTM. The model considers a sequence of five admissions, the indexed one and the previous four. Flat features include features that do not change over a sequence of visits like sex.

As illustrated in Figure B.1, the proposed architecture consists of five streams, four come from embedding layers to handle diagnosis codes, medications at discharge as well as prescribed at history, and lab names with abnormal values. We have a fifth stream for numerical features, specifically age, HF disease age, and administrative variables. An LSTM layer (with 128 units) is used in the model for sequential modeling. The flat features and the output of the LSTM layer are feed into two dense layers with 50 and 10 units respectively, these layers are used as weighting layers before concatenating all streams and feeding their output into a fully connected network (FCN) that acts as a classifier. The FCN consists of five stacked dense layers with the size of 250, 50, 25, 5 and 1 units, with a dropout layer between every two consecutive dense layers.

The LSTM model expects a sequence of five admissions as an input, for patients who have less than five admissions during the whole considered period, padding visits with zero values where added. The masking mechanism was used in order to inform the model that some input sequences contain padding records that should be ignored completely.

**Table S1**: Baseline characteristics—training and holdout test cohorts

| **Characteristics** | **Total**  **(n=15,612)** | **Training Cohort**  **(n=13,800)** | **Holdout Cohort**  **(n=1,812)** |
| --- | --- | --- | --- |
| Sex: Female (%) | 6,905 (44%) | 6,074 (44%) | 831 (46%) |
| Age, in years (± SD) | 79.1 ± 10.4 | 79.2 ± 10.4 | 79.0 ± 10.5 |
| Duration of HF, in days (± SD) | 1,149.9 ± 1,036.6 | 1,137.8 ± 1,021.5 | 1,241.9 ± 1,140.8 |
| Readmissions within 100-days (%) | 5,597 (36%) | 4,971 (36%) | 626 (35%) |

**Sensitivity-based training**

The idea behind sensitivity-based training is to optimize the sensitivity of the trained models. The adopted criterion is to select the model that has the highest sensitivity while keeping specificity to be at least 0.5. This criterion was used to set the classification threshold while training shallow and deep models, then use threshold obtained during training to evaluate the model performance on both validation and holdout datasets. The two models were trained using K-fold cross-validation for 10 iterations, and we chose the model with the highest sensitivity on validation data from these 10 iterations to be tested on holdout dataset. Splitting data into training and testing cohorts is shown at Table B.1.

**C Specification of Medical Codes**

**Table S2**. Specification of diseases used as comorbidity features according to ICD-codes.

| **Diagnose** | **ICD-10-SE codes** |
| --- | --- |
| Hypertension | I10, I11.0, I13.0, I13.2 |
| Ischemic heart disease | I20-I22, I25.1, I25.2, I25.5, I25.6 |
| Cerebrovascular insult | I60-I66, G45, I69 |
| Valvular heart disease | I34-I39 |
| Peripheral artery disease | I70.2, I73.9, I74.2-9 |
| Atrial fibrillation | I48 |
| Diabetes mellitus | E10-E14 |
| Chronic obstructive pulmonary disease | J44 |
| Chronic kidney disease | N00-N08, N10-N19, N08.3, E10.2, E11.2, E12.2, E13.2, E14.2 |

**Table S3.** Specification of treatment according to ATC-codes

| **Medication** | **ATC-code** | |
| --- | --- | --- |
| **β-blockers** |  | |
| Metoprolol succinate | C07AB02 | |
| Bisoprolol | C07AB07 | |
| Carvedilol | C07AG02 | |
| Nebivolol | C07AB12 | |
|  |  | |
| **ACE-inhibitors** |  | |
| Captopril | C09AA01 | |
| Enalapril | C09AA02 | |
| Lisinopril | C09AA03 | |
| Ramipril | C09AA05 | |
| Trandolapril | C09AA10 | |
|  |  | |
| **Angiotensin receptor blockers** |  |  |
| Candesartan | C09CA06 | |
| Losartan | C09CA01 | |
| Valsartan | C09CA03 | |
| **Angiotensin receptor neprilysin inhibitors** | C09DX04 | |
| **Mineralocorticoid receptor antagonists** | C03DA | |
| **Loop diuretics** | C03C | |
| **Digoxin** | C01AA05 | |
| **SGLT-2** | A10BK | |

**Table S4.** ICD-10-SE codes reported by LSTM explanation model

| **Specification** | **ICD-10-SE** | |
| --- | --- | --- |
| **In situ neoplasms** |  | |
| Anemia, unspecified | D.649 | |
|  |  | |
| **Diseases of the nervous system** |  | |
| Paraparesis, unspecified | G.822 | |
|  |  | |
| **Diseases of the circulatory system** |  | |
| Essential hypertension | I.109 | |
| Nonrheumatic aortic valve diseases | I.350 | |
| Heart failure, Insufficientia cordis, Left heart failure | I.501 | |
| Heart failure, unspecified | I.509 | |
|  |  | |
| **Diseases of the musculoskeletal system and connective tissue** | | |
| Spinal stenosis, Caudal stenosis | M.480 | |
| Osteomyelitis, unspecified | M.869 | |
|  |  | |
| **Diseases of the genitourinary system** |  |  |
| Chronic renal failure, unspecified | N.189 | |
|  |  | |
| **Symptoms, signs and abnormal clinical and laboratory findings** | | |
| Other chest pain, Anterior chest-wall pain | R.073 | |
| Difficulty swallowing, Dysphagia | R.139 | |
| **Factors influencing health status and contact with health services** | | |
| Personal history of long-term (current) use of anticoagulants | Z.921 | |

**D SHAP Explanations**

**Figure S3.** SHAP summary for Shallow (CatBoost) showing the global explanation of the model using the top 28 features. The summary plot combines feature importance with feature effects on model’s prediction, such that the position on the y-axis is determined by the feature importance and on the x-axis by the SHAP value. The color represents the value of the feature from low to high in cohort data. For example, lower values of CCI, previous readmissions, and number of ED visits are associated with decreasing readmission risk.

**
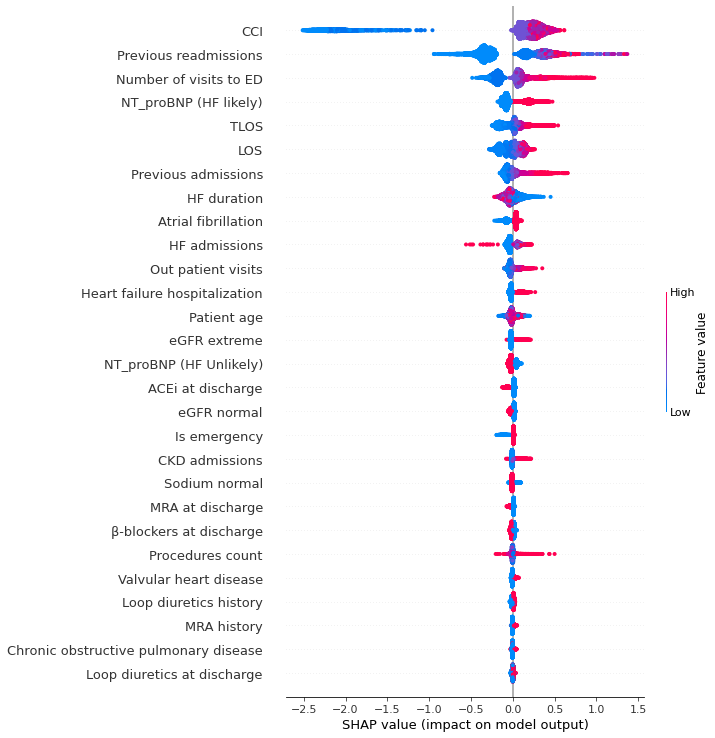
**

**Abbreviations**: ED (emergency department); LOS (length of stay in this admission); TLOS (total length of stay at hospital during the lookback period); CCI (charlson comorbidity index); ACEi (ACE-inhibitors); MRA (Mineralocorticoid receptor antagonists); ARB (Angiotensin receptor blocker).

**Figure S4.** SHAP summary for the Deep (LSTM) showing the global explanation of the model using the top 28 features. LSTM model uses a sequence of five admissions, the one at index and four previous ones as history. Like CatBoost, lower value of previous readmissions decreases the readmission risk. Yet, here the effect of number of ED visits is different from CatBoost model. The features below belong to diagnostic codes, lab values, and some administrative features; no medications appear among top 28 features.


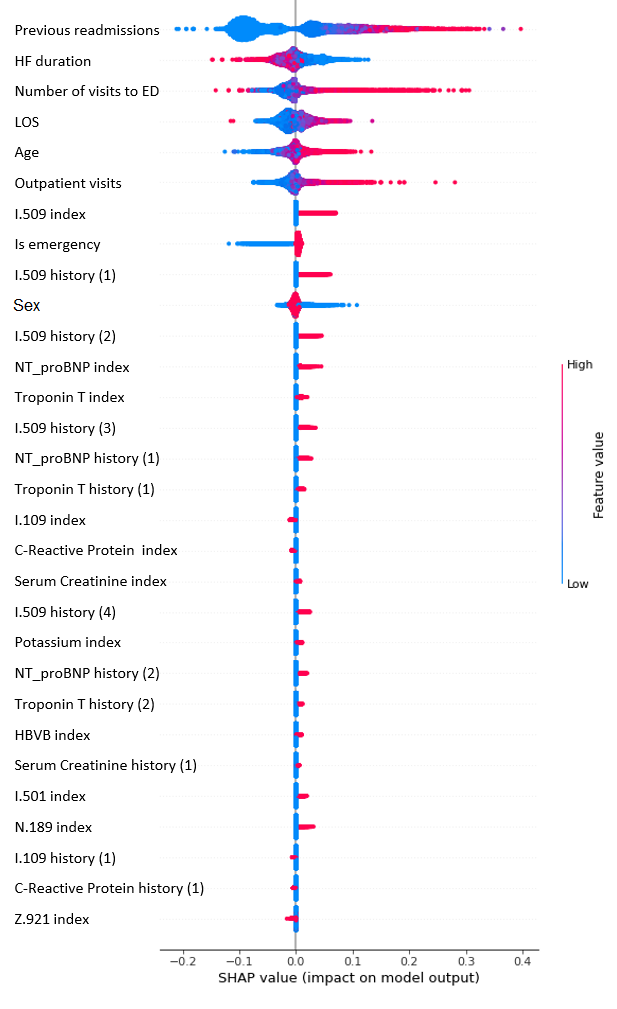


**Abbreviations**: index: represents the index admission, history (1): one visit before index,
history (2): two visits before index, etc.

**Figure S5**. Local explanations for a readmission case correctly classified by the Shallow (CatBoost) model. Features are sorted on y-axis according to their importance. Blue bars indicate that the features are decreasing the readmission risk, on the other hand, red bars show how much features are contributing to increase the readmission risk. Interestingly, both models show NT-proBNP and agree that excluding HF Unlikely value is increasing the readmission risk.

| 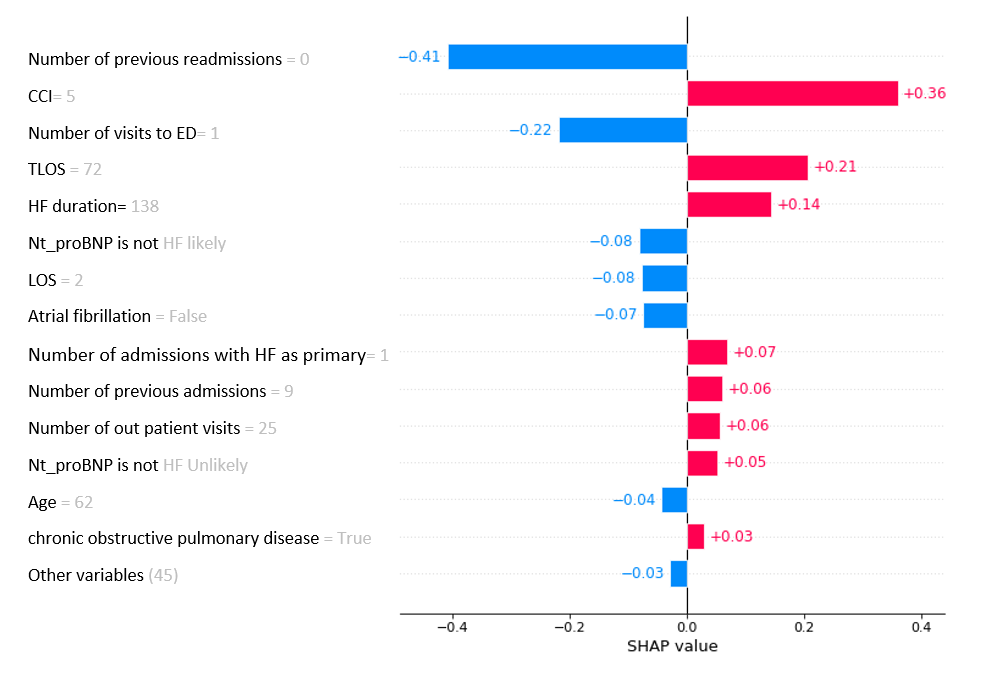   1. Shallow (CatBoost) model |
| --- |
| 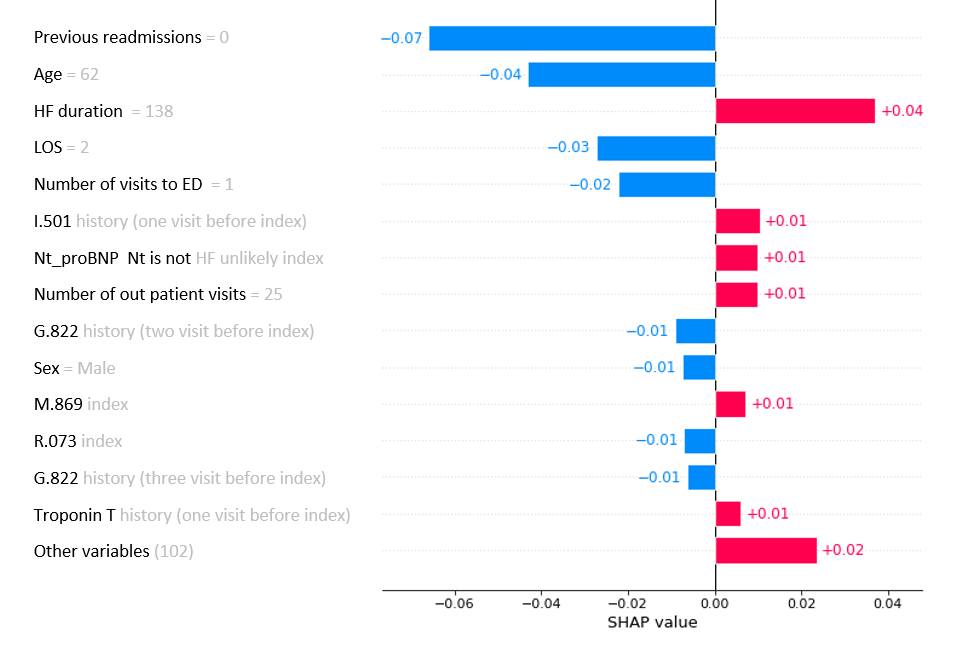   1. Deep (LSTM) model |

**Figure S6**. Local explanations for a readmission case correctly classified by Deep (LSTM) model. Features are sorted on y-axis according to their importance. Blue bars indicate that the features are decreasing the readmission risk, on the other hand, red bars show how much features are contributing to increase the readmission risk. Two models show NT-proBNP, yet disagree on its effect on increasing the readmission risk.

| **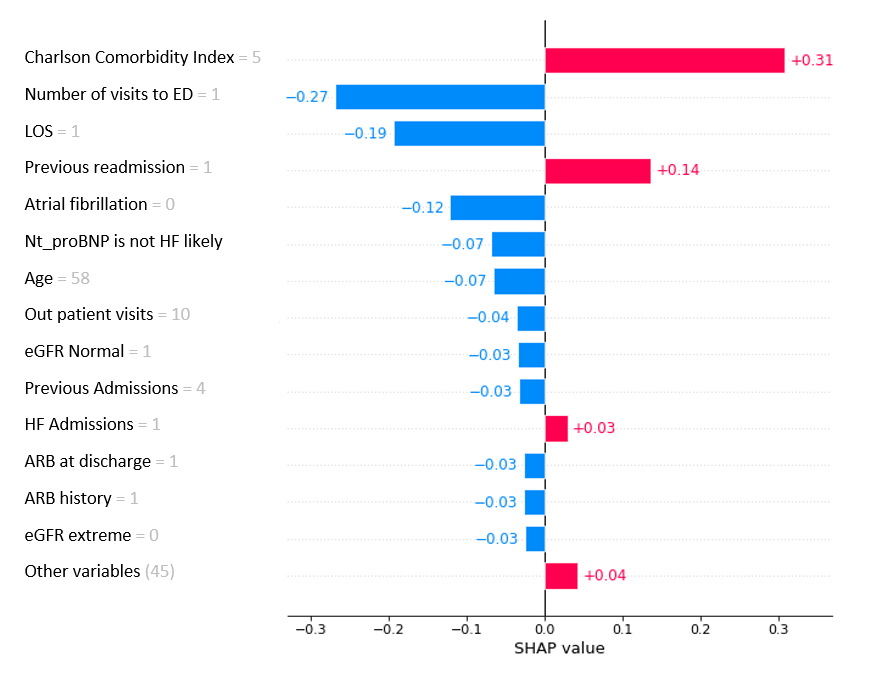**  (a) Shallow (CatBoost) Model |
| --- |
| **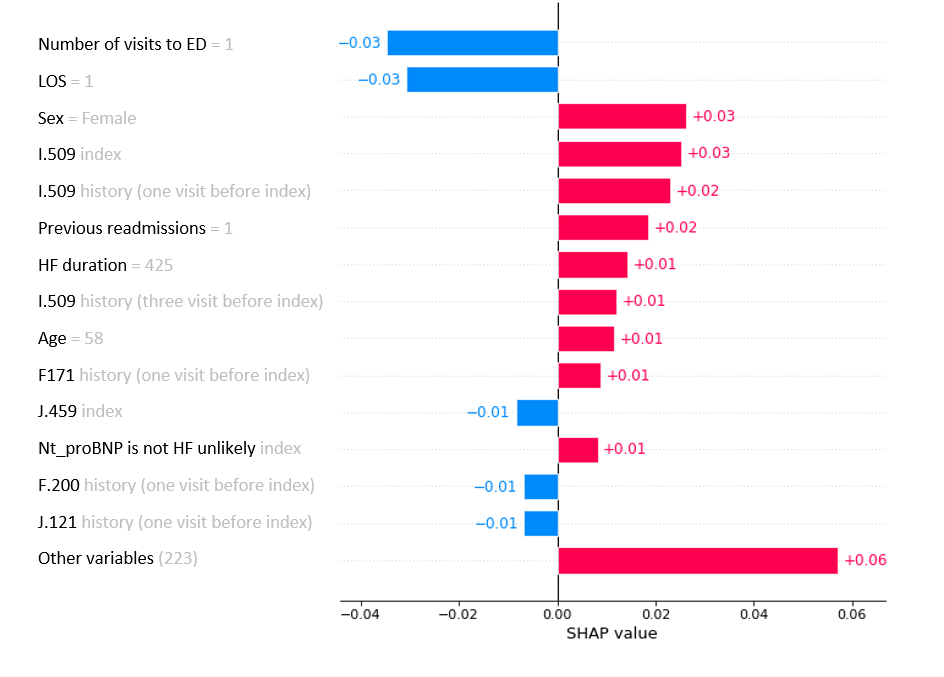**  (b) Deep (LSTM) model |
